# Assessment of the Diagnostic Utility of Serum MicroRNA Classification in Patients With Diffuse Glioma

**DOI:** 10.1001/jamanetworkopen.2019.16953

**Published:** 2019-12-06

**Authors:** Makoto Ohno, Juntaro Matsuzaki, Junpei Kawauchi, Yoshiaki Aoki, Junichiro Miura, Satoko Takizawa, Ken Kato, Hiromi Sakamoto, Yuko Matsushita, Masamichi Takahashi, Yasuji Miyakita, Koichi Ichimura, Yoshitaka Narita, Takahiro Ochiya

**Affiliations:** 1 Department of Neurosurgery and Neuro-oncology, National Cancer Center Hospital, Tokyo, Japan; 2 Division of Molecular and Cellular Medicine, National Cancer Center Research Institute, Tokyo, Japan; 3 Toray Industries, Inc, Tebiro, Kamakura, Japan; 4 Dynacom Co, Ltd, Chiba, Japan; 5 Department of Gastrointestinal Medical Oncology, National Cancer Center Hospital, Tokyo, Japan; 6 Department of Biobank and Tissue Resources, National Cancer Center Research Institute, Tokyo, Japan; 7 Division of Brain Tumor Translational Research, National Cancer Center Research Institute, Tokyo, Japan; 8 Department of Molecular and Cellular Medicine, Tokyo Medical University, Tokyo, Japan

## Abstract

**Importance:**

A blood-based screening tool for detecting diffuse glioma is necessary to improve clinical outcomes.

**Objectives:**

To establish models using serum microRNAs to distinguish patients with diffuse glioma from control individuals without cancer (the Glioma Index) and to differentiate glioblastoma (GBM), primary central nervous system lymphoma (PCNSL), and metastatic brain tumors (the 3-Tumor Index).

**Design, Setting, and Participants:**

This retrospective, case-control diagnostic study included 157 patients with diffuse glioma and 109 patients with central nervous system (CNS) diseases other than diffuse glioma diagnosed from August 1, 2008, through May 1, 2016, and 314 sex- and age-matched controls without cancer. Samples of patients with diffuse glioma and controls were randomly divided into training and validation set 1, and those of patients with CNS diseases other than diffuse glioma were allocated to an exploratory set. Samples of patients with GBM, PCNSL, and metastatic brain tumors were randomly divided into training and validation set 2. Data were analyzed from April 1, 2018, to March 31, 2019.

**Main Outcomes and Measures:**

The expression of 2565 microRNAs was assessed, and the diagnostic performance was evaluated by calculating the area under the receiver operating characteristics curve (AUC), sensitivity, specificity, and accuracy.

**Results:**

A total of 580 patients were included in the analysis (309 [53.3%] male; median age, 57 years [range, 10-87 years]). In training set 1, 100 patients with diffuse glioma (median age, 56 years [range, 14-87 years]; 55 male [55.0%]) were compared with 200 control patients (median age, 56 years [range, 14-87 years]; 105 male [52.5%]), and the Glioma Index was constructed using 3 microRNAs (miR-4763-3p, miR-1915-3p, and miR-3679-5p). In validation set 1, the AUC was 0.99 (95% CI, 0.99-1.00); sensitivity, 0.95 (95% CI, 0.89-1.00); and specificity, 0.97 (95% CI, 0.93-1.00). The Glioma Index classified 39 of 42 PCNSL samples (92.9%) and 25 of 28 metastatic brain tumor samples (89.3%) as positive and 2 of 2 spinal tumors (100%) as negative in the exploratory set. In training set 2, 68 patients with GBM, 34 with PCNSL, and 23 with metastatic brain tumor were compared, and the 3-Tumor Index was constructed using 48 microRNAs. The 3-Tumor Index had an accuracy of 0.80, positively detecting 16 of 17 GBM samples (94.1%), 4 of 5 metastatic brain tumor samples (80.0%), and 4 of 8 PCNSL samples (50.0%) in validation set 2.

**Conclusions and Relevance:**

This study appears to have identified promising serum microRNA combinations for detecting diffuse glioma and for assessing histologic features of brain tumors.

## Key Points

**Question:**

Can serum microRNAs be used to detect diffuse glioma and to differentiate glioblastoma, primary central nervous system lymphoma, and metastatic brain tumors?

**Findings:**

In this case-control diagnostic study of 266 patients with brain or spinal tumors and 314 control patients without cancer, the Glioma Index, constructed using 3 microRNAs, distinguished patients with diffuse glioma from controls with high sensitivity (0.95) and specificity (0.97). The 3-Tumor Index, constructed using 48 microRNAs, positively detected 16 of 17 glioblastomas (94.1%), 4 of 5 metastatic brain tumors (80.0%), and 4 of 8 primary central nervous system lymphomas (50.0%).

**Meaning:**

This study appears to have identified promising microRNA combinations for detecting diffuse glioma and for distinguishing histologic findings in brain tumors.

## Teaser

This diagnostic study establishes and assesses models to detect diffuse glioma and to differentiate glioblastoma, primary central nervous system lymphoma, and metastatic brain tumors.

## Introduction

Diffuse gliomas are the most common primary malignant brain tumors, with an incidence of 1.32 to 5.73 cases per 100 000 adults.^1^ They are diagnosed histologically based on the World Health Organization 2016 brain tumor classification, which integrates histopathologic diagnosis with molecular features.^2^ Glioblastoma (GBM) is the most malignant diffuse glioma, with a 5-year survival rate of approximately 15%.^3^

Standard methods for detecting glioma include neuroradiological examinations such as computed tomography and magnetic resonance imaging. Although these methods are highly sensitive and reliable, their routine use is limited by their cost and inconvenience. Early detection of cancer using screening tools, such as mammography for breast cancer, can improve patient outcomes^4^; however, there are no screening methods for diffuse glioma. Therefore, a screening test for the detection of diffuse glioma with low invasiveness appears to be urgently needed.

Several recent studies investigated the role of circulating microRNAs (miRNAs) as diagnostic biomarkers. MicroRNAs are noncoding RNAs constituting 19 to 24 nucleotides, and they serve as hubs in gene regulatory networks by controlling numerous targets via RNA silencing and posttranscriptional regulation of gene expression.^5^ MicroRNAs are involved in many biological activities, including cancer development. Circulating miRNAs are stable,^6^ and prolonged storage at room temperature, freezing, and thawing has minimal effects on miRNA expression levels.^7^ Circulating miRNA tests are less invasive than other methods and are therefore good candidates for cancer screening tests.

We used serum samples from 580 patients to identify promising miRNAs for the detection of diffuse gliomas. We also explored the potential of miRNA profiles to discriminate among GBM, primary central nervous system lymphoma (PCNSL), and metastatic brain tumor.

## Methods

### Study Population

The study was approved by the institutional review board of the National Cancer Center Hospital (NCCH) and the Research Committee of Medical Corporation Shintokai Yokohama Minoru Clinic. Written informed consent was obtained from all participants. The study followed the Standards for Reporting of Diagnostic Accuracy (STARD) reporting guideline.

Serum samples were obtained from patients who underwent surgery for suspected brain or spinal tumors at the Department of Neurosurgery and Neuro-oncology of the NCCH (n = 215) or who were referred to NCCH after undergoing surgery elsewhere (n = 51) from August 1, 2008, through May 1, 2016. Serum samples were registered and stored at −20 °C in the National Cancer Center Biobank. Patients who were referred to NCCH after undergoing surgery elsewhere were included only if they had residual tumor at the time of referral to NCCH. Tumor diagnoses were based on the World Health Organization 2016 classification^2^ (eMethods 1 in the Supplement). The 266 patients with central nervous system (CNS) disease included 157 with diffuse gliomas, 13 with glial tumors other than diffuse glioma (7 ependymomas, 3 pilocytic astrocytomas, 2 anaplastic gangliogliomas, and 1 unclassified glioma), 42 with PCNSL, 28 with metastatic brain tumor, 22 with benign brain tumors (19 meningiomas, 2 hemangiopericytomas, and 1 intracranial schwannoma), 2 with spinal schwannomas, 1 with trauma, and 1 with infarction.

The 314 control patients without cancer included 157 patients with benign diseases and no cancer treated at the NCCH from 2008 through 2016 (noncancer sample 1); serum samples from these patients were stored at −20 °C and matched for sex and age with those of 157 patients with diffuse glioma. Another 157 healthy individuals older than 35 years were seen for medical checkup. Serum samples from these patients were collected at the Yokohama Minoru Clinic in 2014 (noncancer sample 2). These serum samples were stored at −80 °C and matched by sex and age with those of the 157 patients with diffuse glioma. The use of samples collected and stored under different conditions minimized the effects of differences in collection and storage conditions.

### MiRNA Extraction and Expression Analysis

Total RNA was extracted from 300-μL serum samples using RNA extraction reagent (3D-Gene System; Toray Industries, Inc) and concentrated. Fluorescent labeling of RNA was performed using a miRNA labeling kit (3D-Gene System). RNA was hybridized to a human miRNA oligo chip (3D-Gene System) designed to detect 2565 miRNA sequences in miRBase, release 21 (http://www.mirbase.org/), and the chip was scanned (3D-Gene System). MicroRNAs with signals higher than the background signal were selected in advance (positive call), and background signals were subtracted from each positive-call miRNA signal. Only preprocessed positive-call miRNAs were used for subsequent analyses. To normalize signals across microarrays, miRNA signals were divided by the mean signals of internal control miRNAs (miR-149-3p, miR-2861, and miR-4463) that were stably detected in more than 500 serum samples.^8^ To identify robust miRNAs, miRNAs with normalized signal values of greater than 64 intensity units in more than 50% of samples in each group were selected. Microarray data were obtained in accordance with the Minimum Information About a Microarray Experiment (MIAME) guidelines. Data sets were submitted to the National Center for Biotechnology Information Gene Expression Omnibus database under accession number GSE 139031.

### Statistical Analysis

**Figure 1.**
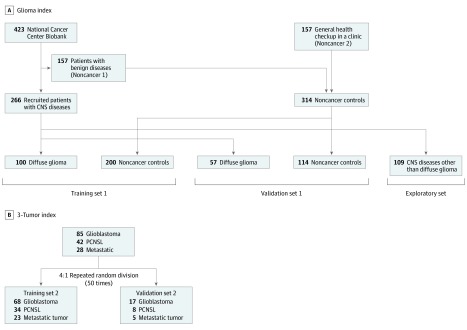
Flowchart of the Development of the Glioma Index and the 3-Tumor Index A, For the Glioma Index, samples were divided into training set 1, validation set 1, and the exploratory set. Noncancer control samples were collected from the National Cancer Center Biobank (noncancer 1 controls) and the general population undergoing routine health checkup at a clinic in Yokohama, Japan (noncancer 2 controls). B, For the 3-Tumor Index, samples were divided randomly into 2 groups (4:1, training set 2 and validation set 2) to develop models for discriminating between glioblastoma (GBM), primary central nervous system (CNS) lymphoma (PCNSL), and metastatic brain tumors.

Data were analyzed from April 1, 2018, to March 31, 2019. To develop models for discrimination between patients with diffuse gliomas and control patients without cancer, samples from the patient and control groups were randomly divided (100:57) into training set 1 and validation set 1. Training set 1 was used to construct discrimination models, and validation set 1 was used to validate the discrimination models. Other brain tumors (ependymomas, pilocytic astrocytomas, gangliogliomas, PCNSL, metastatic brain tumor, meningiomas, and schwannomas), spinal tumors, and trauma and infarction cases were allocated to the exploratory set to investigate the ability of the model to identify gliomas among these cases (Figure 1A).

Two-group discrimination models were constructed using Fisher linear discriminant analysis and leave-1-out cross-validation in training set 1 (a flowchart is shown in eMethods 2 in the Supplement). Cutoff values for discrimination models were set at 0 based on the Youden index. The best discrimination model (ie, the model showing maximum accuracy using the minimum number of miRNAs) was selected in training set 1. In validation set 1, diagnostic sensitivity, specificity, accuracy, and area under the receiver operating characteristics curve (AUC) were calculated.

To develop a 3-group discrimination model to distinguish among GBM, PCNSL, and metastatic brain tumor, samples were randomly divided (4:1) into training set 2 and validation set 2. Three-group discrimination models were constructed by combining the 2-group discrimination models. First, 3 types of 2-group discrimination models were constructed as follows: (1) GBM vs others (PCNSL and metastatic brain tumor); (2) PCNSL vs others (GBM and metastatic brain tumor); and (3) metastatic brain tumor vs others (GBM and PCNSL). In this case, the discrimination models were constructed using the logistic least absolute shrinkage and selection operator regression analysis with 10-fold cross-validation. The random division was repeated 50 times, and fifty 2-group discrimination models were constructed. Next, fifty 3-group discrimination models were constructed by combining models 1 and 2, 2 and 3, or 1 and 3. Finally, 1 representative 3-group discrimination model was selected, and the diagnostic accuracy was tested in validation set 2 (a flowchart is shown in eMethods 3 in the Supplement).

Fisher linear discriminant analysis and logistic least absolute shrinkage and selection operator regression analysis were performed using the following packages in R, version 3.1.2 (R Project for Statistical Computing): compute.es, version 0.2-4; glmnet, version 2.0-3; hash, version 2.2.6; MASS, version 7.3-45; mutoss, version 0.1-10; and pROC, version 1.8. Unsupervised clustering and heatmap generation were performed using Pearson correlation in the Ward method for linkage analysis, and principal component analysis was performed using Genomics Suite, version 6.6 (Partek). Differences in characteristics between 2 or 3 groups were evaluated using the unpaired *t* test (continuous variables), Pearson χ^2^ test (categorical variables), and SPSS, version 22 (IBM Japan). The limit of statistical significance for all analyses was defined as a 2-sided *P* < .05.

## Results

### Characteristics of Patients With CNS Diseases and Controls

The characteristics of the 580 participants (309 [53.3%] male; 271 [46.7%] female; median age, 57 years [range, 10-87 years]) in training set 1, validation set 1, and the exploratory set are shown in Table 1. Training set 1 (n = 300) consisted of 100 patients with diffuse glioma (median age, 56 years [range, 14-87 years]; 55 male [55.0%] and 45 female [45.0%]) and 200 control patients without cancer (median age, 56 years [range, 14-87 years; 105 male [52.5%] and 95 female [47.5%]). Validation set 1 (n = 171) consisted of 57 patients with diffuse glioma (median age, 54 years [range, 17-84 years]; 34 male [59.6%] and 23 female [40.4%]) and 114 control patients (median age, 56 years [range, 21-85 years]; 58 male [50.9%] and 56 female [49.1%]). The exploratory set (n = 109) consisted of patients with CNS diseases other than diffuse glioma (Table 1). No statistically significant differences were found in age and sex between patients with diffuse glioma and control patients in training set 1 or validation set 1 (eTable 1 in the Supplement).

**Table 1.** Patient Characteristics^a^

Characteristic	Training Set 1 (n = 300)	Validation Set 1 (n = 171)	Exploratory Set (n = 109)	*P* Value
**Patients With Diffuse Glioma**				
Total	100 (33.3)	57 (33.3)	NA	NA
Age, median (range), y	56 (14-87)	54 (17-84)	NA	.46
Sex				
Male	55 (55.0)	34 (59.6)	NA	.62
Female	45 (45.0)	23 (40.4)	NA	
Histologic finding				
Diffuse astrocytoma	14 (14.0)	11 (19.3)	NA	NA
IDH-Mut	8 (8.0)	8 (14.0)	NA	.06
IDH-WT	5 (5.0)	0	NA	
NOS	1 (1.0)	3 (5.3)	NA	
Oligodendroglioma	3 (3.0)	3 (5.3)	NA	
IDH-Mut, 1p/19q codeletion	3 (3.0)	2 (3.5)	NA	.27
NOS	0	1 (1.8)	NA	
Anaplastic astrocytoma	21 (21.0)	10 (17.5)	NA	
IDH-Mut	10 (10.0)	2 (3.5)	NA	.17
IDH-WT	4 (4.0)	5 (8.8)	NA	
NOS	7 (7.0)	3 (5.3)	NA	
Anaplastic oligodendroglioma	6 (6.0)	4 (7.0)	NA	NA
IDH-Mut, 1p/19q codeletion	6 (6.0)	4 (7.0)	NA	NA
Glioblastoma	56 (56.0)	29 (50.9)	NA	NA
IDH-Mut	3 (3.0)	1 (1.8)	NA	.59
IDH-WT	43 (43.0)	25 (43.9)	NA	
NOS	10 (10.0)	3 (5.3)	NA	
**Patients With CNS Disease Other Than Diffuse Glioma**				
Total	NA	NA	109 (100)	NA
Age, median (range), y	NA	NA	65 (10-85)	NA
Sex				
Male	NA	NA	57 (52.3)	NA
Female	NA	NA	52 (47.7)	NA
Histologic finding				
Glial tumors other than diffuse glioma (ependymoma, pilocytic astrocytoma, etc)	NA	NA	13 (11.9)	NA
Primary CNS lymphoma	NA	NA	42 (38.5)	NA
Metastatic brain tumor	NA	NA	28 (25.7)	NA
Benign brain tumor (meningioma, schwannoma, etc)	NA	NA	22 (20.2)	NA
Spinal schwannomas	NA	NA	2 (1.8)	NA
Trauma	NA	NA	1 (0.9)	NA
Infarction	NA	NA	1 (0.9)	NA
**Controls Without Cancer**				
Total	200 (100)	114 (100)	NA	NA
Age, median (range), y	56 (14-85)	56 (21-85)	NA	.94
Sex				
Male	105 (52.5)	58 (50.9)	NA	.78
Female	95 (47.5)	56 (49.1)	NA	
Sample collection				
National Cancer Center Biobank	100 (50.0)	57 (50.0)	NA	NA
General health checkup in a clinic	100 (50.0)	57 (50.0)	NA	

Abbreviations: CNS, central nervous system; IDH-Mut, IDH1/2 mutation; IDH-WT, IDH1/2 wild-type; NA, not applicable; NOS, not otherwise specified.

^a^ Unless otherwise specified, data are presented as number (percentage).

### Development of the Glioma Index

**Figure 2.**
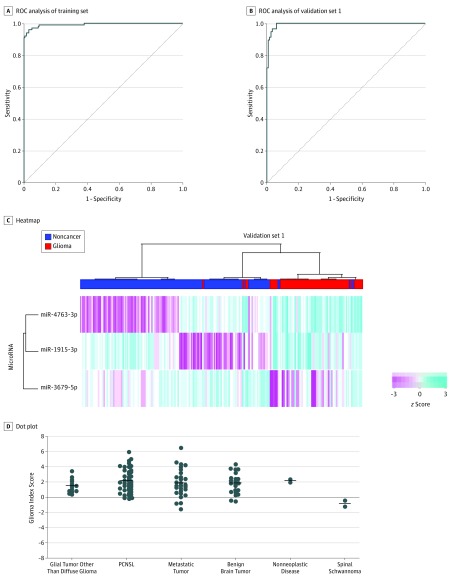
Development and Validation of the Glioma Index A, In training set 1 of the Glioma Index, sensitivity was 0.96 (95% CI, 0.92-1.00); specificity, 0.97 (95% CI, 0.95-0.97); and area under the receiver operating characteristic (ROC) curve, 0.99 (95% CI, 0.99-1.00). B, In validation set 1 of the Glioma Index, sensitivity was 0.95 (95% CI, 0.89-1.00); specificity, 0.97 (95% CI, 0.93-1.00); and area under the ROC curve, 0.99 (95% CI, 0.99-1.00). C, The diffuse glioma and noncancer control samples in validation set 1 were plotted using unsupervised hierarchical clustering analysis with a heat map for the Glioma Index. *z* Scores indicate how many SDs a point is away from the mean of its data set. D, Dot plot of the Glioma Index in the exploratory set. The sensitivity for glial tumors other than diffuse glioma (n = 13) was 1.00; primary central nervous system lymphoma (PCNSL) (n = 42), 0.93; metastatic brain tumor (n = 28), 0.89; benign brain tumor (n = 22), 0.91; nonneoplastic disease (trauma and infarction) (n = 2), 1.00; and spinal schwannoma (n = 2), 0. The horizontal bar indicates the mean.

Among 2565 miRNAs, 365 had normalized signal values greater than 64 intensity units in more than 50% of samples in each group. Six miRNAs were removed based on miRBase, release 22; ultimately, 359 miRNAs were analyzed in this study. Table 2 shows the best combination models of 1 to 5 miRNAs in training set 1. For the Glioma Index, we selected a model based on 3 miRNAs (miR-4763-3p, miR-1915-3p, and miR-3679-5p) that achieved high accuracy (0.97; 95% CI, 0.95-0.99); the AUC indicated no improvement in models with miRNA combinations. The Glioma Index had a sensitivity of 0.96 (95% CI, 0.92-1.00), a specificity of 0.97 (95% CI, 0.95-0.97), and an AUC of 0.99 (95% CI, 0.99-1.00) (Figure 2A). The Glioma Index was calculated as follows: (2.09406 × miR-4763-3p) + (1.35369 × miR-1915-3p) + (−0.378659 × miR-3679-5p) − 32.11268. The AUC of a single miRNA to distinguish diffuse glioma from noncancer controls ranged from 0.63 to 0.92 (eFigure 1 in the Supplement).

**Table 2.** Best Combination Models of MicroRNAs to Detect Diffuse Glioma in Training Set 1

No. of miRNAs	Model Candidates	Training Set 1				
		Sensitivity (95% CI)	Specificity (95% CI)	Accuracy (95% CI)	AUC (95% CI)	*P* Value
1	(1.42387 × miR-1225-3p) − 10.2225	0.93 (0.88-0.98)	0.83 (0.77-0.88)	0.86 (0.82-0.90)	0.95 (0.93-0.97)	NA
2	(1.21875 × miR-1225-3p) + (1.62781 × miR-1227-5p) − 25.2598	0.90 (0.84-0.96)	0.97 (0.95-0.99)	0.95 (0.92-0.97)	0.97 (0.96-0.99)	.006 (vs 1-miRNA model)
3	(2.09406 × miR-4763-3p) + (1.35369 × miR-1915-3p) + (–0.378659 × miR-3679-5p) − 32.11268	0.96 (0.92-1.00)	0.97 (0.95-0.97)	0.97 (0.95-0.99)	0.99 (0.99-1.00)	.01 (vs 2-miRNA model)
4	(1.78027 × miR-4763-3p) + (1.24861 × miR-1915-3p) + (–0.352535 × miR-3679-5p) + (0.295729 × miR-6729-3p) −30.12238	0.96 (0.92-1.00)	0.98 (0.96-1.00)	0.97 (0.95-0.99)	1.00 (0.99-1.00)	.15 (vs 3-miRNA model)
5	(2.03114 × miR-4763-3p) + (1.32272 × miR-1915-3p) + (–0.427725 × miR-3679-5p) + (0.494549 × miR-4741) + (–0.126557 × miR-204-3p) −34.1707	0.96 (0.92-1.00)	1.00 (1.00-1.00)	0.99 (0.97-1.00)	0.99 (0.99-1.00)	.83 (vs 4-miRNA model)

Abbreviations: AUC, area under the receiver operating characteristic curve; miRNAs, microRNAs; NA, not applicable.

The diagnostic performance of the Glioma Index was tested in validation set 1, yielding a sensitivity of 0.95 (95% CI, 0.89-1.00), specificity of 0.97 (95% CI, 0.93-1.00), and AUC of 0.99 (95% CI, 0.99-1.00) (Figure 2B). The unsupervised hierarchical clustering analysis (depicted by a heat map in Figure 2C) and principal component analysis (eFigure 2A in the Supplement) demonstrated that the Glioma Index effectively differentiated diffuse glioma from noncancer controls. The discrimination performance of the Glioma Index did not vary among diffuse astrocytoma, oligodendroglioma, anaplastic astrocytoma, anaplastic oligodendroglioma, and GBM (eFigure 2B in the Supplement).

Next, we tested the diagnostic performance of the Glioma Index using the exploratory set. As shown in Figure 2D, this model classified 13 of 13 glial tumors other than diffuse glioma (100%), 39 of 42 PCNSL samples (92.9%), 25 of 28 metastatic brain tumor samples (89.3%), and 20 of 22 benign brain tumors (90.9%) as positive. Nonneoplastic cases, including trauma and infarction, were also classified as positive. Two spinal schwannoma cases were classified as negative.

### Development of the 3-Tumor Index

Next, we examined the ability of serum miRNA profiles to distinguish histologic features of tumors. We focused on GBM, PCNSL, and metastatic brain tumor because, although these tumors appear as contrast-enhanced lesions on computed tomography and magnetic resonance imaging, these modalities cannot always discriminate among the 3 types of tumors.

The study cohort consisted of 85 patients with GBM, 42 with PCNSL, and 28 with metastatic brain tumor, with no differences in age and sex between patients with these 3 tumors (eTable 2 in the Supplement). To develop a 3-group discrimination model, we randomly divided GBM, PCNSL, and metastatic brain tumor cases into training set 2, consisting of 68 patients with GBM, 34 with PCNSL, and 23 with metastatic brain tumor; and validation set 2, consisting of 17 with GBM, 8 with PCNSL, and 5 with metastatic brain tumor (Figure 1B).

Fifty 2-group discrimination models using 50 patterns of random division were initially generated in GBM vs others (PCNSL and metastatic brain tumor), PCNSL vs others (GBM and metastatic brain tumor), and metastatic brain tumor vs others (GBM and PCNSL). In the GBM vs others discrimination model, the mean number of miRNAs was 22.6 (95% CI, 20.2-25.0), and the mean AUC was 0.91 (95% CI, 0.89-0.92) (eFigure 3A in the Supplement). Similarly, in discrimination models of PCNSL vs others, the mean number of miRNAs was 16.0 (95% CI, 13.1-19.0) and the mean AUC was 0.86 (95% CI, 0.84-0.88); for metastatic brain tumor vs others, the mean number of miRNAs was 21.1 (95% CI, 18.2-24.0) and the mean AUC was 0.97 (95% CI, 0.96-0.98) (eFigure 3B and C in the Supplement).

To construct the 3-group discrimination model, 2 of the 2-group discrimination models were combined, and their accuracy was compared. In the combination of GBM vs others and PCNSL vs others, the mean accuracy was 0.76 (95% CI, 0.64-0.89) (eFigure 3D in the Supplement). In the combination of PCNSL vs others and metastatic brain tumor vs others, the mean accuracy was 0.80 (95% CI, 0.69-0.95) (eFigure 3E in the Supplement). In the combination of GBM vs others and metastatic brain tumor vs others, the mean accuracy was 0.79 (95% CI, 0.70-0.91) (eFigure 3F in the Supplement).

Finally, we selected a representative model (3-Tumor Index) to discriminate GBM, PCNSL, and metastatic brain tumor in training set 2 using 48 miRNAs (eTable 3 in the Supplement). This 3-Tumor Index had an accuracy of 0.80 in training set 2 (Table 3) and classified 16 of 17 GBM samples (94.1%) and 4 of 5 metastatic brain tumor samples (80.0%) as positive in validation set 2. However, 4 of 8 PCNSL samples (50.0%) were misdiagnosed as GBM (Table 3).

**Table 3.** Cross Tabulation of the 3-Tumor Index in Training and Validation Set 2

Test Result	True Diagnosis, No./Total No. (%)		
	GBM	PCNSL	Metastatic Brain Tumor
Training set			
GBM	62/68 (91.2)	17/34 (50.0)	1/23 (4.3)
PCNSL	6/68 (8.8)	17/34 (50.0)	0/23
Metastatic brain tumor	0/68	0/34	21/23 (91.3)
Not determined	0/68	0/34	1/23 (4.3)
Validation set			
GBM	16/17 (94.1)	4/8 (50.0)	1/5 (20.0)
PCNSL	1/17 (5.9)	4/8 (50.0)	0/5
Metastatic brain tumor	0/17	0/8	4/5 (80.0)

Abbreviations: GBM, glioblastoma; PCNSL, primary central nervous system lymphoma.

## Discussion

In this case-control diagnostic study, we comprehensively analyzed 580 serum samples and 2565 miRNAs, including samples from 157 patients with diffuse glioma, using a highly sensitive method of miRNA microarray analysis. We then developed the Glioma Index, which discriminates diffuse glioma from noncancer controls, and the 3-Tumor Index, which discriminates among GBM, PCNSL, and metastatic brain tumor.

Previous studies^9,10,11,12,13^ reported different miRNA signatures that distinguish patients with glioma from healthy individuals with varying diagnostic performance. Zhi et al^14^ evaluated 739 miRNAs in serum samples from 90 patients with astrocytoma and identified 9 miRNAs (miR-15-5p, miR-16-5p, miR-19a-3p, miR-19b-3p, miR-20a-5p, miR-106a-5p, miR-130a-3p, miR-181b-5p, and miR-208a-3p) as potential biomarkers capable of distinguishing astrocytomas from controls (AUC, 0.9722). Zhou et al^15^ reviewed 28 articles investigating miRNA-based glioma diagnosis and reported overall sensitivity of 85%, specificity of 90%, and AUC of 93% for these models. Although these studies suggest that miRNAs could be useful for distinguishing patients with glioma from healthy individuals, a complete consensus has not been reached to date. This inconsistency may be attributed to heterogeneity in study design (patients with gliomas or astrocytomas vs healthy controls), sample type (ie, serum vs plasma, whole blood vs exosomes), miRNA coverage, and analytical techniques. The present method using the microarray excluded the 9 miRNAs (miR-15-5p, miR-16-5p, miR-19a-3p, miR-19b-3p, miR-20a-5p, miR-106a-5p, miR-130a-3p, miR-181b-5p, and miR-208a-3p) detected by polymerase chain reaction by Zhi et al^14^ because of low expression levels. This discrepancy may be attributed to the different method of detecting miRNAs. The Glioma Index developed in our study was based on 3 miRNAs (miR-4763-3p, miR-1915-3p, and miR-3679-5p) and had a sensitivity of 0.95 (95% CI, 0.89-1.00), specificity of 0.97 (95% CI, 0.93-1.00), and AUC of 0.99 (95% CI, 0.99-1.00) in validation set 1. We believe these statistics represent excellent diagnostic power and provide definitive and reliable discrimination between diffuse gliomas and noncancer controls.

We also investigated whether the Glioma Index could discriminate diffuse gliomas from other intracranial diseases in the exploratory set; however, we found it difficult to discriminate between diffuse gliomas and other brain tumors. We speculate that the differences between diffuse gliomas and noncancer controls detected by the Glioma Index were not the same as those between diffuse gliomas and other brain tumors. However, from a clinical point of view, the ability of the Glioma Index to distinguish brain tumors from noncancer controls suggests that it may be useful as a screening tool for detecting intracranial diseases during medical visits. There are currently no population-based screening tests for brain tumors, and the Glioma Index appears to be a promising candidate screening tool that could be used before referring patients for computed tomographic or magnetic resonance imaging examination. Furthermore, the Glioma Index showed high sensitivity not only in patients with GBM, but also in those with lower-grade glioma (100% for grade 2 diffuse astrocytoma and 100% for grade 2 oligodendroglioma) (eFigure 2B in the Supplement), suggesting the possibility of detecting diffuse glioma at the early stage. In the context of the clinical treatment of diffuse gliomas, early detection would enable tumor resection before the tumor has undergone malignant transformation or infiltrated adjacent brain tissues. Therefore, 2-step screening combined with blood-based screening test and neuroradiological examination could potentially improve patient outcomes.

The Glioma Index included 3 miRNAs: miR-4763-3p, miR-1915-3p, and miR-3679-5p. The serum levels of miR-4763-3p and miR-1915-3p were higher in patients with diffuse glioma than in controls, suggesting that these miRNAs are oncogenic. By contrast, the level of miR-3679-5p was lower in patients with diffuse glioma than in controls, suggesting that the miRNA functions as a tumor suppressor. Although the roles of these miRNAs in glioma remain unclear, their cancer-related functions were suggested in previous studies.^16,17,18^ Expression of miR-4763-3p is induced by oxidative stress in hepatocellular carcinoma cells, and its putative target genes are associated with cell growth arrest and apoptosis.^16^ Guo et al^17^ showed that miR-1915-3p is upregulated in breast cancer and promotes the migration and proliferation of breast cancer cells. miR-3679-5p is significantly downregulated in malignant pancreatic tumors relative to healthy controls or benign pancreatic tumors, although its function remains unknown, to our knowledge.^18^

We also demonstrated that miRNA levels may be able to be used to distinguish among GBM (sensitivity, 0.94), PCNSL (sensitivity, 0.50), and metastatic brain tumor (sensitivity, 0.80). The Glioma Index consisted of 3 miRNAs and was highly sensitive and specific, whereas the 3-Tumor Index consisted of 48 miRNAs and had low sensitivity for PCNSL. This finding suggests that distinguishing between different histologic features of intracranial tumors is more complex than differentiating brain tumors from noncancer controls. Clinically, discriminating among these 3 tumor types using serum miRNAs is important because the treatment strategies for each type are different. Developing blood-based diagnostic methods that can distinguish among GBM, PCNSL, and metastatic brain tumor without a biopsy may enable timely initiation of treatment, thereby avoiding neurosurgical risks. Although the discriminatory performance for PCNSL was not satisfactory, the fact that miRNAs could identify GBM and metastatic brain tumor is encouraging. Thus, miRNA-based diagnosis could complement neuroradiology to support clinical decision-making.

### Limitations

This study had several limitations. First, it was performed using retrospectively collected samples. Consequently, sample handling conditions before microarray analysis, such as the interval between centrifugation and storage and the storage temperature, were not strictly regulated and sometimes differed between samples. Although miRNAs are more stable than messenger RNA, various processes can affect their levels in serum.^19,20^ Second, we lacked an external validation cohort to test the performance of the Glioma Index and the 3-Tumor Index. We have initiated a prospective, multicenter study by collecting serum samples using standard operating procedures and will assess the generalizability of our data prospectively using this independent cohort. Third, this study was a case-control study, which does not allow calculation of positive and negative predictive values. However, because of the rarity of brain tumors, conducting prospective cohort studies is difficult in terms of cost and time. We believe the case-control study design is adequate for collecting large volumes of brain tumor samples in a short time and for exploring the proof-of-concept. Fourth, the noncancer control samples were not the optimal control population, despite being sex and age matched with the diffuse glioma group. Specimens in noncancer sample 1 were collected from patients with benign diseases and stored at −20 °C in the National Cancer Center Biobank. Specimens in noncancer sample 2 were collected from healthy individuals undergoing medical checkup and stored at −80 °C at a different institution. The use of these samples as noncancer controls may have affected the results because of the differences in patient background or storage conditions, potentially leading to the overestimation of specificity. To minimize these biases, we used samples from 2 cohorts with different backgrounds and storage conditions. Fifth, the present cohort was limited to Japanese patients. Because genetic background can affect miRNA expression, further studies are needed to confirm the performance of the present indexes in different racial/ethnic cohorts.

## Conclusions

In summary, we identified promising serum miRNA combinations for detecting diffuse glioma with high sensitivity and specificity and to distinguish histologic features of brain tumors. The present data suggest that evaluation of circulating miRNAs is suitable for primary screening of brain tumors. The development of blood-based diagnostic strategies for the detection of brain tumors could contribute to improved patient outcomes. Further studies are needed to confirm the present observations.

## Notes

Supplement. **eMethods 1.** Molecular Diagnosis of Diffuse Glioma **eMethods 2.** Algorithm: Procedure for Constructing 2-Group Discrimination Models **eMethods 3.** Algorithm: Procedure for Constructing 3-Group Discrimination Models **eTable 1.** Differences in Age and Sex Distribution Between Diffuse Glioma and Noncancer Controls **eTable 2.** Differences in Age and Sex Distribution Between Glioblastoma, Primary Central Nervous System Lymphoma, and Metastatic Brain Tumors **eTable 3.** The 48 miRNAs of the 3-Tumor Index for Discriminating Among Glioblastoma, Primary Central Nervous System Lymphoma, and Metastatic Brain Tumors **eFigure 1.** Diagnostic Utility of a Single miRNA to Distinguish Glioma From Noncancer **eFigure 2.** Validation of the Glioma Index **eFigure 3.** Development of the 3-Tumor Index **eReferences.**
