# Supplementary material for: Assessment of the Diagnostic Utility of Serum MicroRNA Classification in Patients With Diffuse Glioma
Source: JAMA Netw Open. 2019 Dec 6;2(12):e1916953. doi: 10.1001/jamanetworkopen.2019.16953 (PMC6902771; doi:10.1001/jamanetworkopen.2019.16953)
Supplement: Supplement. — eMethods 1. Molecular Diagnosis of Diffuse Glioma eMethods 2. Algorithm: Procedure for Constructing 2-Group Discrimination Models eMethods 3. Algorithm: Procedure for Constructing 3-Group Discrimination Models eTable 1. Differences in Age and Sex Distribution Between Diffuse Glioma and Noncancer Controls eTable 2. Differences in Age and Sex Distribution Between Glioblastoma, Primary Central Nervous System Lymphoma, and Metastatic Brain Tumors eTable 3. The 48 miRNAs of the 3-Tumor Index for Discriminating Among Glioblastoma, Primary Central Nervous System Lymphoma, and Metastatic Brain Tumors eFigure 1. Diagnostic Utility of a Single miRNA to Distinguish Glioma From Noncancer eFigure 2. Validation of the Glioma Index eFigure 3. Development of the 3-Tumor Index eReferences. [file jamanetwopen-2-e1916953-s001.pdf]

Supplementary Online Content

Ohno M, Matsuzaki J, Kawauchi J, et al. Assessment of the diagnostic utility of serum microRNA classification in patients with diffuse glioma. *JAMA Netw Open*. 2019;2(12):e1916953. doi:10.1001/jamanetworkopen.2019.16953

eMethods 1. Molecular Diagnosis of Diffuse Glioma
eMethods 2. Algorithm: Procedure for Constructing 2-Group Discrimination Models
eMethods 3. Algorithm: Procedure for Constructing 3-Group Discrimination Models
eTable 1. Differences in Age and Sex Distribution Between Diffuse Glioma and Noncancer Controls
eTable 2. Differences in Age and Sex Distribution Between Glioblastoma, Primary Central Nervous System Lymphoma, and Metastatic Brain Tumors
eTable 3. The 48 miRNAs of the 3-Tumor Index for Discriminating Among Glioblastoma, Primary Central Nervous System Lymphoma, and Metastatic Brain Tumors
eFigure 1. Diagnostic Utility of a Single miRNA to Distinguish Glioma From Noncancer
eFigure 2. Validation of the Glioma Index
eFigure 3. Development of the 3-Tumor Index
eReferences.

This supplementary material has been provided by the authors to give readers additional information about their work.

eMethods 1. Molecular Diagnosis of Diffuse Glioma

Determination of *IDH1/2* mutation status

IDH1/2 mutation status was determined as previously described[1]. Briefly, total DNA was extracted from frozen tissue samples or paraffin-embedded specimens using a DNeasy Blood & Tissue kit (Qiagen, Maryland, USA). Polymerase chain reaction (PCR) was performed to amplify a 129 base pair (bp) fragment of *IDH1* containing codon 132 or a 150 bp fragment of *IDH2* containing codon 172. The PCR products were purified using the QIAquick PCR Purification kit (Qiagen, Maryland, USA). DNA sequencing of the *IDH1/2* gene was performed using the same primers used for PCR[1].

1p and 19q status by multiplex ligation-dependent probe amplification analysis

The SALSA P088 kit (MRC, Amsterdam, Netherlands) containing 16 1p probes (6 probes at 1p36), 8 19q probes, and 21 control probes specific to other chromosomes, including 2 probes for 19p, was used. Information regarding the probe sequences and ligation sites can be found at http://www.mlpa.com. Multiplex ligation-dependent probe amplification analysis was performed as previously described[2]. 1p36 or 19q deletions were considered present when five of six markers for 1p36 and five of eight markers for 19q in each chromosome arm had normalized ratios <0.75.

© 2019 Ohno M et al. *JAMA Network Open.*

eMethods 2. Algorithm: Procedure for Constructing 2-Group Discrimination Models

Notation:

N: Number of candidate sets of miRNAs

M: Maximum combined number of miRNAs in each candidate set

T: Total number of miRNAs

Score: Accuracy = (TP + TN) / (TP + FP + FN + TN), where each variable is defined as follows:

	Cancer	Non-cancer
Prediction positive	TP: number of true positives	FP: number of false positives
Prediction negative	FN: number of false negatives	TN: number of true negatives

Procedure for constructing two-group discrimination models

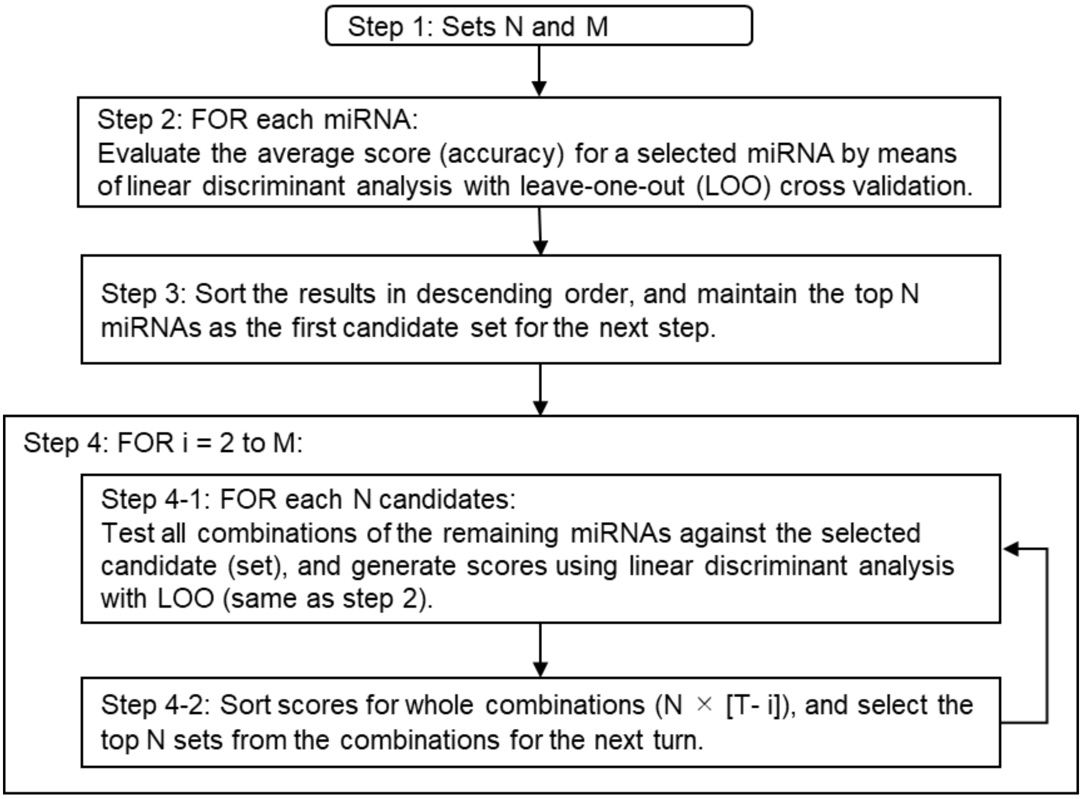

© 2019 Ohno M et al. *JAMA Network Open.*

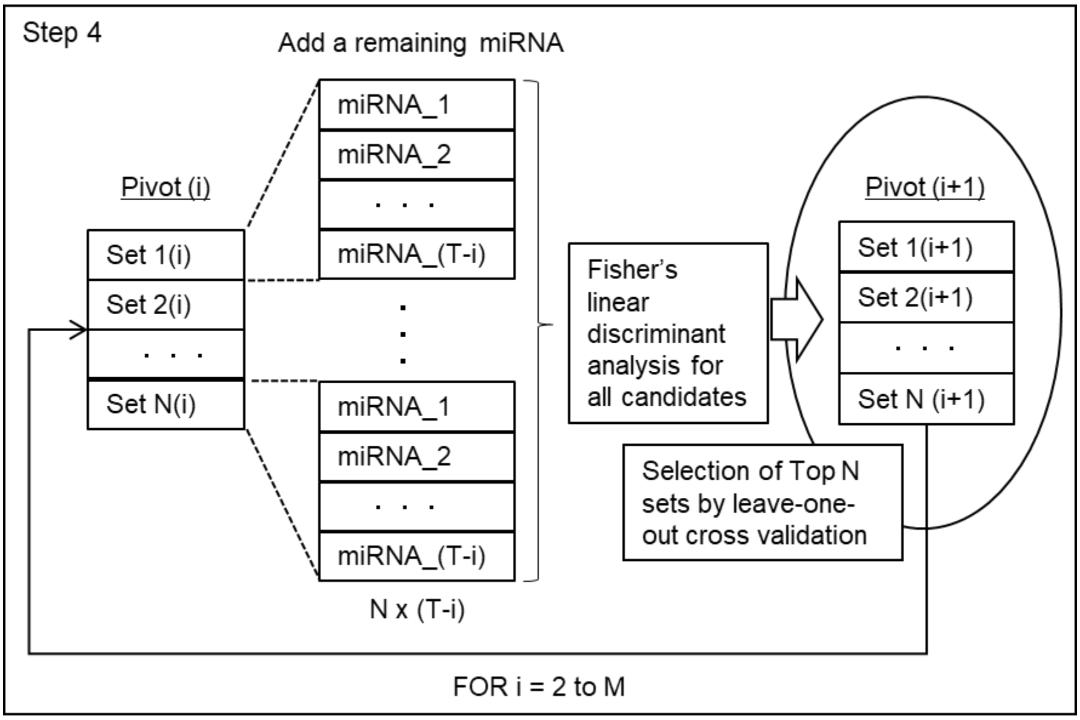

© 2019 Ohno M et al. *JAMA Network Open.*

eMethods 3. Algorithm: Procedure for Constructing 3-Group Discrimination Models

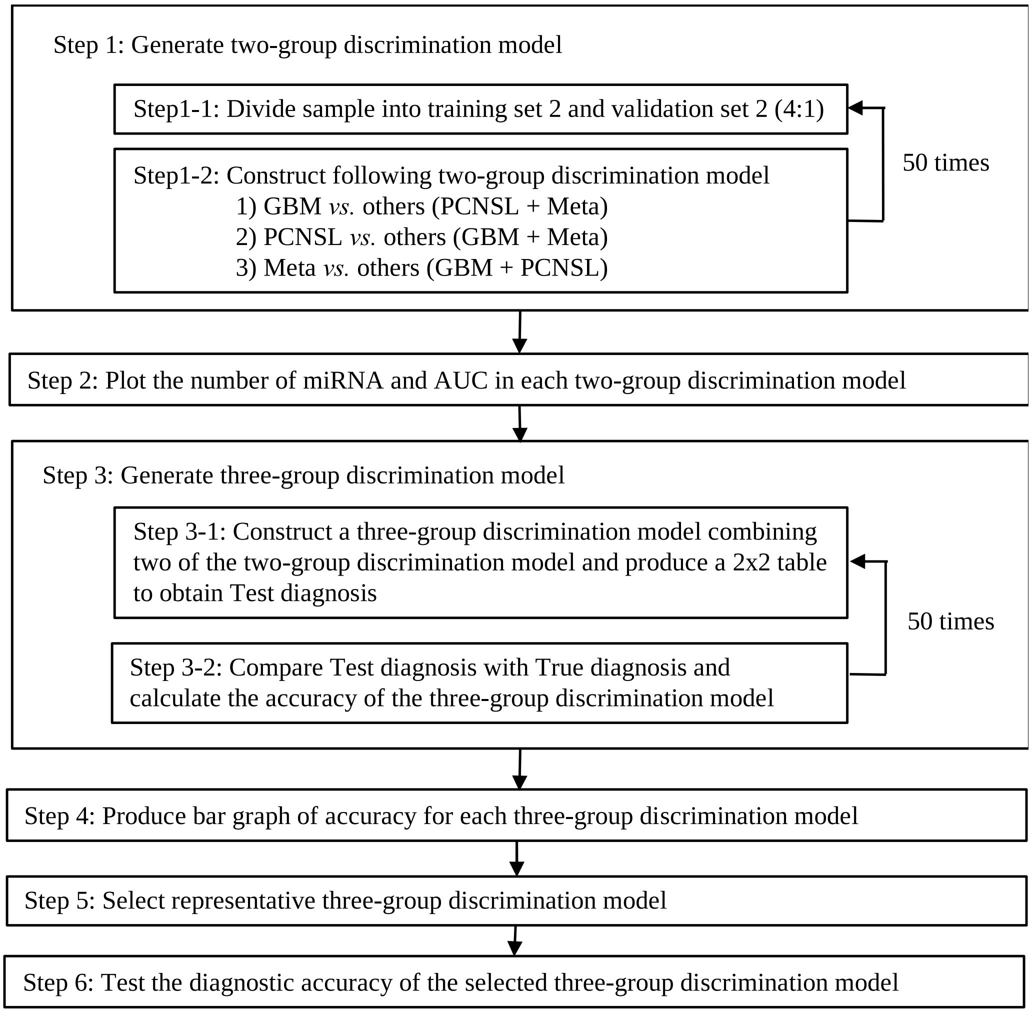

© 2019 Ohno M et al. *JAMA Network Open.*

eTable 1. Differences in Age and Sex Distribution Between Diffuse Glioma and Noncancer Controls

	Diffuse glioma	Non-cancer control	p-value
Training set 1			
Total No. (%) of controls	100 (100)	200 (100)	
Age, median (range), y	56 (14-87)	56 (14-87)	0.76
Sex, No (%)			
Male	55 (55)	105 (53)	0.71
Female	45 (45)	95 (47)	
Validation set 1			
Total No. (%) of controls	57 (100)	114 (100)	
Age, median (range), y	54 (17-84)	56 (21-85)	0.27
Sex, No (%)			
Male	34 (60)	58 (51)	0.33
Female	23 (40)	56 (49)	

© 2019 Ohno M et al. *JAMA Network Open.*

eTable 2. Differences in Age and Sex Distribution Between Glioblastoma, Primary Central Nervous System Lymphoma, and Metastatic Brain Tumors

	Glioblastoma	Primary central nervous system lymphoma	Metastatic brain tumors	p-value
Total No. (%) of controls	85 (100)	42 (100)	28 (100)	
Age, median (range), y	64 (17-87)	68 (26-83)	65 (24-76)	0.35
Sex, No (%)				
Male	50 (59)	28 (67)	15 (54)	0.52
Female	35 (41)	14 (33)	13 (46)	

© 2019 Ohno M et al. *JAMA Network Open.*

eTable 3. The 48 miRNAs of the 3-Tumor Index for Discriminating Among Glioblastoma, Primary Central Nervous System Lymphoma, and Metastatic Brain Tumors

PCNSL *vs.* others		Meta *vs.* others	
miRNA	coefficient	miRNA	coefficient
miR-6805-3p	0·190489650252051	miR-106a-3p	-0·169331585033951
miR-7975	0·0517934180028152	miR-8089	-0·112387073327149
miR-150-3p	0·0665429842438766	miR-486-3p	-0·262743085618485
miR-1260b	0·0798221872319207	miR-342-5p	-0·358975372637574
miR-4463	-0·534594030509306	miR-4745-5p	0·931617802602443
miR-6515-3p	-0·042686416640624	miR-4436b-5p	0·186040255268863
miR-6766-3p	0·0463998153269611	miR-1343-5p	0·99465235140153
miR-6877-5p	0·114096181422196	miR-211-3p	-0·043161525668922
(Intercept)	0·448392266883874	miR-6802-5p	-0·0490187784742703
		miR-5196-5p	0·182874895847875
		miR-150-3p	-0·0857222032212309
		miR-4771	0·356212246362562
		miR-4708-3p	-0·120221341715771
		miR-532-3p	0·0971112759086939
		miR-4656	0·735777831882568
		miR-2116-3p	-0·0495061766171574
		miR-365a-5p	-0·351490105773066
		miR-933	0·147348446578719
		miR-6124	0·172233229713192
		miR-3620-5p	0·110134738673348
		miR-4258	-0·844141800239268
		miR-4463	-5·06699399218911
		miR-6070	-0·438010489511108
		miR-7113-3p	0·157441741158861
		miR-602	0·136609511995946

© 2019 Ohno M et al. *JAMA Network Open.*

		miR-4476	-0·09895499489422
		miR-6752-5p	0·604317562770886
		miR-5195-3p	-0·399759936495029
		miR-527, miR-518a-5p	-0·177548780512667
		miR-4633-3p	-0·0638715916223451
		miR-4758-5p	-0·905128414759015
		miR-6515-3p	0·279852663573411
		miR-4706	-0·128531860847318
		miR-92a-2-5p	0·0667828090931865
		miR-6721-5p	0·0146798829604962
		miR-4454	0·452555086047712
		miR-4449	-0·108063588826539
		miR-1233-5p	0·393322000540513
		miR-4787-3p	-0·831389608058257
		miR-6796-3p	0·464305123117306
		miR-4313	0·357508573387304
		miR-1225-5p	0·102500142942516
		miR-1224-3p	0·0478254585863892
		(Intercept)	30·1186134638357
LASSO: Least absolute shrinkage and selection operator, PCNSL: Primary central nervous system lymphoma, Meta: Metastatic brain tumor			

© 2019 Ohno M et al. *JAMA Network Open.*

eFigure 1. Diagnostic Utility of a Single miRNA to Distinguish Glioma From Noncancer

Receiver operating characteristic (ROC) curves for the detection of patients with diffuse glioma using miR-4763-3p, miR-1915-3p, and miR-3679-5p in validation set 1. The area under the curve (AUC) values for miR-4763-3p, miR-1915-3p, and miR-3679-5p were 0.92, 0.79, and 0.63, respectively.

A

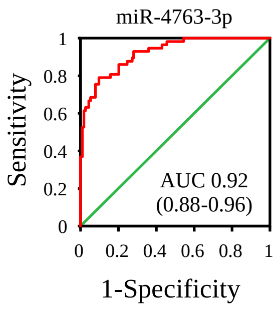

B

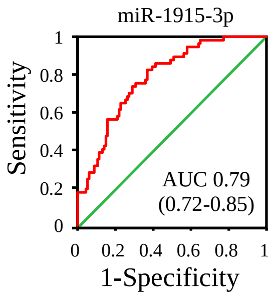

C

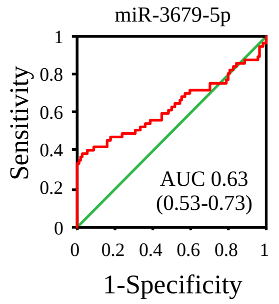

© 2019 Ohno M et al. *JAMA Network Open.*

eFigure 2. Validation of the Glioma Index

A) **Principal component analysis**

Principal component analysis of the Glioma Index, showing clear separation between diffuse gliomas and non-cancer controls using validation set 1. N = 57 for diffuse glioma, and 114 for non-cancer.

B) **Dot plot of the Glioma Index in diffuse glioma and non-cancer controls**

The Glioma Index discriminated diffuse gliomas from non-cancer controls irrespective of diffuse glioma subtype in validation set 1. Each diagnostic accuracy (%) is included. N = 11 for diffuse astrocytoma, grade II (DA); 3 for oligodendroglioma, grade II (OL); 10 for anaplastic astrocytoma, grade III; 4 for anaplastic oligodendroglioma, grade III (AO); 29 for glioblastoma, grade IV (GBM); 57 for Non-cancer 1 (NC1); and 57 for Non-cancer 2 (NC2).

A

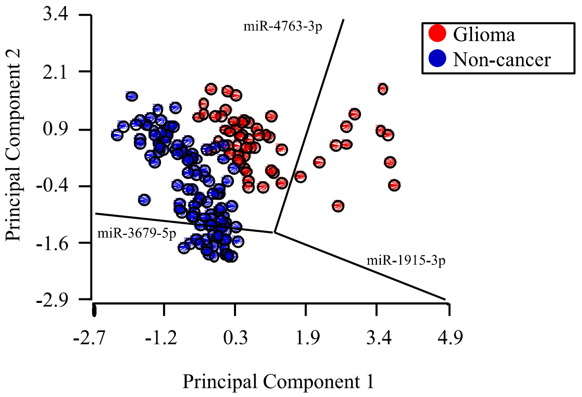

B

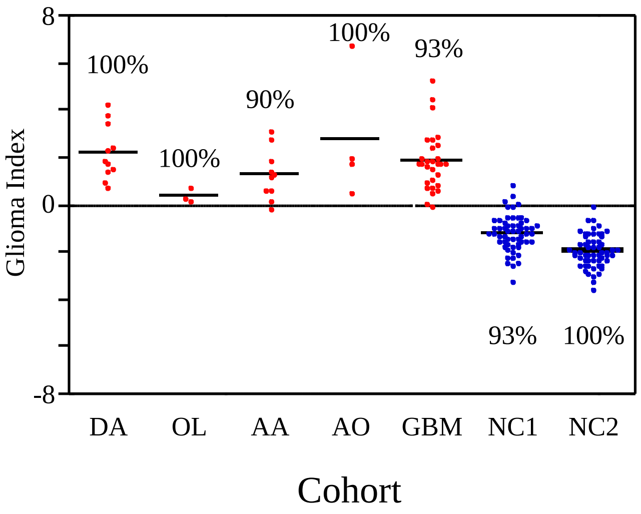

© 2019 Ohno M et al. *JAMA Network Open.*

eFigure 3. Development of the 3-Tumor Index

A-C) Relationship between the area under the curve (AUC) and the number of miRNAs in 50 different two-group discrimination models in training set 2

The X and Y axes indicate the number of miRNAs and the AUC, respectively. The 50 different two-group discrimination models were plotted as open circles. The red open circle indicates the average number of miRNAs and the error bar indicates standard deviation.

A) In the GBM vs. others discrimination model, the average number of miRNAs was 22.6 and the mean AUC was 0.91.

B) In the PCNSL vs. others discrimination model, the average number of miRNAs was 16.0 and the mean AUC was 0.86.

C) In the Meta vs. others discrimination model, the average number of miRNAs was 21.1 and the mean AUC was 0.97.

A

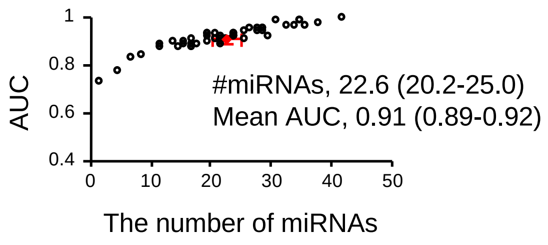

B

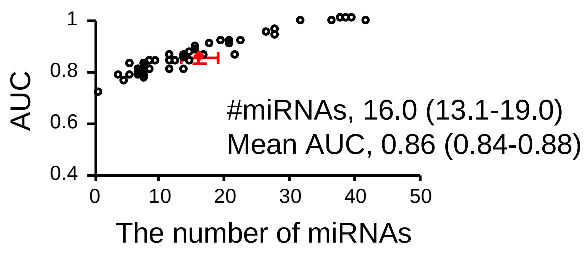

C

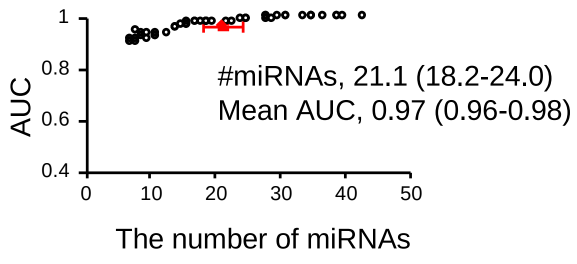

© 2019 Ohno M et al. *JAMA Network Open.*

D-F) Combination of two-group discrimination models to produce a 2 × 2 table for analyzing the model accuracy in training set 2.

The X and Y axes indicate the accuracy and the frequency of the model, respectively. Each mean diagnostic accuracy (%) is included.

D) In the combination of GBM vs. others and PCNSL vs. others, the mean accuracy was 0.76.

E) In the combination of PCNSL vs. others and Meta vs. others, the mean accuracy was 0.80.

F) In the combination of GBM vs. others and Meta vs. others, the mean accuracy was 0.79.

D)

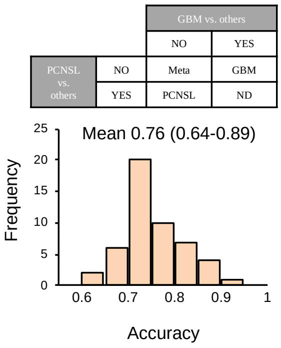

E)

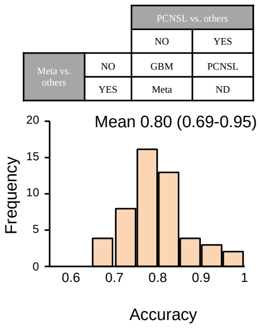

F)

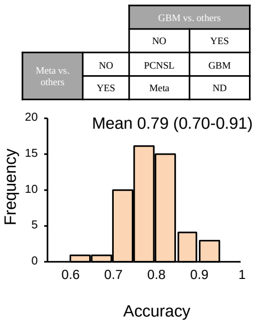

© 2019 Ohno M et al. *JAMA Network Open.*

eReferences

1. Arita H, Narita Y, Matsushita Y, et al. Development of a robust and sensi- tive pyrosequencing assay for the detection of IDH1/2 mutations in gliomas. *Brain Tumor Pathol* 2015;32:22–30.
2. Okita Y, Narita Y, Miyakita Y, et al. IDH1/2 mutation is a prognostic mark- er for survival and predicts response to chemotherapy for grade II gliomas concomitantly treated with radiation therapy. Int J Oncol 2012;41: 1325–36.

© 2019 Ohno M et al. *JAMA Network Open.*
